# Factors associated with willingness to participate in clinical trials: a nationwide survey study

**DOI:** 10.1186/s12889-014-1339-0

**Published:** 2015-01-17

**Authors:** Sang Hui Chu, Eun Jung Kim, Seok Hee Jeong, Geu Lee Park

**Affiliations:** Nursing Policy and Research Institute, Biobehavioral Research Center, College of Nursing, Yonsei University, Seoul, Republic of Korea; Division of Nursing, College of Medicine, Hallym University, Chuncheon, Gangwon-do Republic of Korea; College of Nursing, Research Institute of Nursing Science, Chonbuk National University, 567 Baekje-daero, deokjin-gu, Jeonju-si, Jeollabuk-do 561-756 Republic of Korea; Cardiovascular Center, Severance Hospital, Yonsei University Health System, Seoul, Republic of Korea

**Keywords:** Clinical trial, Perception, Awareness, Patient participation

## Abstract

**Background:**

This study was conducted to investigate awareness of clinical trials (CTs) including perceptions of favorable feelings about, necessity for, and safety of CTs, the ultimate beneficiary of CTs and the factors associated with willingness to participate in CTs among the general population in South Korea.

**Methods:**

A cross sectional survey study was conducted in a randomly selected national sample of 1,515 Korean.

**Results:**

Perception toward CTs was measured using a scale from 0 (strongly disagree) to 10 (strongly agree). Respondents readily understood the necessity for CTs (M = 7.27, SD = 2.15); had moderately favorable feelings (M = 5.32, SD = 2.31) toward CTs and felt that these CTs were moderately safe (M = 4.71, SD = 1.90). Twenty-five percent of the respondents answered that they would be willing to participate in a CT in the future. Perception of the ultimate benefits of CTs, awareness, favorable feelings, safety, and necessity regarding CTs were identified as significant predictors of willingness to participate in CTs.

**Conclusion:**

An awareness of CTs and the perceptions toward CTs were associated with general public willingness to participate in a CT. Findings from this study can be used in planning outreach and recruitment strategies, and to understand the predictors of CT participation.

## Background

South Korea has become one of the most active countries performing industry-sponsored trials under the trend of globalization [1]. Participant recruitment is one of the most important steps that determine the success of a trial [2-4]. The perception and attitude of participants toward clinical trials (CTs) are a key determinant in successful recruitment and retention. One systematic review reported that the most common factor that motivates people to participate in CTs is altruism [5]. However, many previous studies on this issue have been conducted in Western countries or with patients with specific ethnicities, such as Caucasians, African-Americans, or Latinos [6-10]. Due to the social and cultural differences between Eastern and Western countries, it is difficult to directly generalize outcomes obtained in the West to the people of Asia, including Koreans.

Over the past decade, the number of CTs in Asian countries has increased rapidly due to important evidence based on ethnic diversity and cost-effectiveness in clinical trials. However, less is known about CT participation in Asians [11], and studies that included large samples from the general Asian public remain quite limited. Therefore, the investigation of what the Korean people know and how they feel about CTs is the first step toward implementing these outcomes to improve participation in clinical research in Asian countries and caring present and future participants.

We conducted this study to identify the public awareness of CTs and the factors affecting willingness to participate in clinical trials in Korea.

## Methods

### Study design and participants

This was a descriptive study using a face-to-face questionnaire survey method. The target population was the general South Korean population aged 19 years and over. A three-stage, stratified, systematic random sampling method was used to select 1,515 nationally representative participants of interest. The sampling and data collection process in this study was conducted in cooperation with Gallup Korea, which is a specialized marketing research company in Korea, from 7–24 November 2008. The sampling domain was subdivided into ‘strata’ by the size of municipalities. Regions were divided by metropolitan cities (population more than 1,000,000), cities (population between 50,000 and 1,000,000), and rural area (population less than 50,000) based on the Local Autonomy Law of Korea. Metropolitan cities and cities were considered urban. A total of 15 strata were defined (seven metropolitan cities and eight provinces including 40 cities and 10 rural areas). The number of surveys in each stratum was calculated in proportion to the distribution of the household and population of the ‘stratum’ in the area based on 2007 Korea statistics of resident registration, the latest data available. Strata were narrowed to 102 smaller units of administrative districts in urban areas and rural areas such as ‘dong’ for urban areas and ‘eup or myon’ for rural area. The principal investigator (PI) trained the interviewers about the study purpose and procedure before data collection and monitored the data collection process throughout the study period. The sampling error for this study was ±3% at a 95% confidence interval (CI). Ethical approval was obtained from the Institutional Review Board of Severance Hospital of Yonsei University Health System (No. 4-2008-0415).

### Instruments

The investigators developed a questionnaire in collaboration with clinical researchers and clinical research coordinators. The questionnaire included basic demographic information and experience with CT participation; awareness of CTs and source of information of CTs; perceptions on favorable feelings about, necessity for, and safety of CTs; perception of the ultimate beneficiary of CTs; and willingness to participate in CTs.

Awareness of CTs was measured by asking “Have you ever heard about CTs?” Participants were given the following seven choices regarding the sources from which they obtained information about CTs: relatives or friends; mass media, such as TV or radio, newspaper, internet; advertisements for recruitment in hospitals; promotional material; and medical staff. Before assessing the general perception of CTs, interviewers explained CTs to participants using a standardized definition of CTs (any trial or investigation in human subjects to be intended to verify the effects of an investigational drugs or treatments with the object of ascertaining its safety and/or efficacy). Three questions were asked concerning the participants’ perceptions of CTs: favorable feeling, necessity and safety. Each question was measured using an 11-point Likert-type scale. Scores of 0–3, 4–6, and 7–10 were assigned to correspond with low-level, moderate-level, and high-level classes, respectively. We assessed any previous experience with CTs by asking “Have you ever participated in CTs?” The question of perception on the ultimate beneficiary of CTs had four possible answer choices: pharmaceutical companies, hospitals or physicians, patients, and advances in medical science or national economic benefits. The question about willingness to participate in CTs was measured dichotomously by asking “What is the likelihood of your taking part in a clinical trial in the future?” and “If your family member had a disease and needed a new treatment or drug, would you be willing to participate in a clinical trial?” A pilot test of the questionnaire was performed to determine question clarity and average time required for completion.

### Statistical analyses

Descriptive statistics of respondent demographics, awareness of CTs, perceptions of CTs, and willingness to participate in CTs were analyzed. Associations among the variables were evaluated by χ^2^ test, independent t-test, or one-way ANOVA. Multivariate analysis was also conducted by logistic regression. Adjusted odds ratios (ORs) and 95% confidence interval (CI) were calculated by logistic regression methods to examine the effects of the awareness, experience, and perception variables on willingness to participate in CT. All analyses were performed using the Statistical Package for the Social Sciences (SPSS) for Windows, version 15.0 at a level of significance of *p* < .05.

## Results

Demographic data of the 1,515 respondents are shown in Table 1. Males and females were evenly represented in this study (49.7% vs. 50.3%). Overall, 47.5% of the respondents lived in metropolitan cities, 41.1% in small cities, and 11.4% in rural areas. Only 2.1% of the participants had previously participated in CTs.

**Table 1:** **General characteristics of respondents (N = 1,515)**

**Variable**	**Category**	**n**	**%**
Gender	Male	753	49.7
	Female	762	50.3
Age (years)	19-29	295	19.5
	30s	375	24.8
	40s	411	27.1
	50s	250	16.5
	≥60	184	12.1
Education level	≤ High school	958	63.2
	≥ College	557	36.8
Region	Metropolitan cities	720	47.5
	Cities	623	41.1
	Rural area	172	11.4
Household income (1000 won/month)	<2,000	347	22.9
	2,000-2,999	406	26.8
	3,000-3,999	353	23.3
	≥4,000	390	25.7
	Don’t know	19	1.3
Living style	Alone	79	5.2
	With family	1433	94.8
Experience in a clinical trial	No	1114	97.9
	Yes	24	2.1

Missing data were not included.

### Awareness of CTs

When asked whether they had ever heard about CTs, 75.1% of respondents answered in the affirmative. Awareness of clinical trials was significantly more prevalent among male respondents, 30–49 years of age, who were more highly educated and were residents of metropolitan cities or rural areas (Table 2). The five most cited methods from which CT information had been obtained (in descending order) were mass media, such as TV and radio (86%) and newspapers (24.6%), relatives or friends (8.8%), the internet (7.5%), and advertisements in hospitals (4.2%).

**Table 2:** **Respondent awareness of clinical trials (N = 1,515)**

**Variable**		**Yes n (%)**	**χ** ^**2**^	***p***
Gender	Male	594 (78.9)	11.377	.001
	Female	544 (71.4)		
Age (years)	19-29	215 (72.9)	85.792	<.001
	30s	306 (81.6)		
	40s	348 (84.7)		
	50s	173 (69.2)		
	≥60	96 (52.2)		
Education level	≤ High school	692 (72.2)	11.576	.001
	≥ College	446 (80.1)		
Region	Metropolitan cities	565 (78.5)	13.100	.001
	Cities	438 (70.3)		
	Rural area	135 (78.5)		
Household income (1,000 won/month)	<2,000	208 (59.9)	73.604	<.001
	2,000-2,999	337 (83.0)		
	3,000-3,999	284 (80.5)		
	≥4,000	301 (77.2)		
Living style	Alone	48 (60.8)	9.115	.003
	With family	1087 (75.1)		

### Perceptions toward CTs

The mean scores for the three categories of favorable feeling, safety, and necessity of CTs were 5.32 (SD = 2.31), 4.71 (SD = 1.90) and 7.27 (SD = 2.15), respectively. The mean score of necessity among younger adults was significantly lower than those of adults in their 30s, 40s, and 50s (*p* < .001). Significant differences were found between residents of metropolitan cities and those of other areas (*p* < .001). Awareness of CTs was significantly associated with favorable feeling, safety, and necessity of CTs. Those who were aware of CTs had significantly higher favorable feeling (*p* < .001), necessity (*p* < .001), and safety (*p* < .001) perceptions toward CTs than the respondents who were not (Table 3). A question regarding the ultimate outcome of CTs indicated that 45% of respondents believed that patients received benefit from CTs. This was followed by pharmaceutical companies (30.6%), advances in medical science (15.6%), and hospitals or physicians (9.0%).

**Table 3:** **Respondent perception of clinical trials (N = 1,515)**

**Variable**	**Category**	**Favorable feeling**			**Safety**			**Necessity**		
		**M (SD)**	**t or F**	***p***	**M (SD)**	**t or F**	***p***	**M (SD)**	**t or F**	***p***
Gender	Male	5.40 (2.34)	1.378	.168	4.70 (1.93)	−0.292	.770	7.36 (2.18)	1.677	.094
	Female	5.24 (2.28)			4.72 (1.86)			7.17 (2.12)		
Age (years)	19 ~ 29	5.00 (2.23)	20.752	.004	4.57 (1.90)	5.984	.156	6.77 (2.16)^a^	26.135	<.001
	30s	5.11 (2.30)			4.64 (1.85)			7.28 (2.30)^b^		a < b
	40s	5.53 (2.22)			4.90 (1.81)			7.49 (1.99)^b^		
	50s	5.54 (2.55)			4.74 (1.95)			7.46 (2.20)^b^		
	≥60	5.49 (2.24)			4.61 (2.10)			7.26 (1.99)		
Education	≤High School	5.37 (2.30)	6.427	.272	4.73 (1.87)	1.205	.563	7.29 (2.14)	1.113	.624
	≥ College	5.23 (2.32)			4.67 (1.95)			7.23 (2.18)		
Region	Large city	5.44 (2.24)	13.682	.077	4.82 (1.97)	9.319	.075	7.00 (2.20)^a^	53.033	<.001
	Small city	5.16 (2.42)			4.58 (1.86)			7.46 (2.16)^b^		a < b
	Rural area	5.37 (2.16)			4.73 (1.70)			7.68 (1.75)^b^		
Household income	<2,000	5.24 (2.41)	8.345	.181	4.69 (1.88)	0.957	.901	7.02 (2.30)	16.105	.007
	2,000-2,999	5.43 (2.20)			4.76 (1.85)			7.37 (1.91)		
	3,000-3,999	5.14 (2.24)			4.65 (2.00)			7.13 (2.24)		
	≥4,000	5.39 (2.41)			4.72 (1.89)			7.44 (2.16)		
Living style	Alone	5.39 (2.30)	0.301	.763	4.66 (1.69)	−0.234	.815	7.23 (2.07)	−0.153	.879
	With family	5.31 (2.31)			4.71 (1.91)			7.27 (2.16)		
Awareness of CT	No	4.66 (2.33)	−6.469	<.001	4.21 (1.86)	−5.910	<.001	6.66 (2.39)	−5.901	<.001
	Yes	5.54 (2.26)			4.87 (1.89)			7.47 (2.03)		
Experience in CT	No	5.51 (2.26)	−2.938	.003	4.88 (1.87)	0.436	.663	7.47 (2.02)	0.834	.404
	Yes	6.88 (2.17)			4.71 (2.44)			7.13 (2.19)		

a < b in post doc test.

### Willingness to participate in CTs

We asked “What is the likelihood of your taking part in a clinical trial in the future?”, 25% of respondents indicated that they would be willing to participate in a CT. When asked, “if your family member had a disease and needed a new treatment or drug, would you be willing to participate in a clinical trial?”, 48.6% of respondents indicated their willingness to participate.

Table 4 shows the associations between variables and willingness to participate in CTs in the univariate analysis. Six variables (perception on ultimate beneficiary of CTs, experience, awareness, favorable feeling, safety, and necessity) showed a significant association with willingness to participate in CTs according to the Chi-squared test (*p* < .001, *p* = .042, *p* < .001, *p* < .001, *p* < .001, and *p* < .001, respectively). Other factors, including gender, age, educational level, resident area, household income, or living style, were not significantly associated with willingness to participate in CTs.

**Table 4:** **Respondent willingness to participate in clinical trials (N = 1,515)**

**Variable**		**n (%)**	***x*** ^***2***^	***p***
Gender	Male	204/753 (27.1)	3.665	.056^a^
	Female	174/762 (22.8)		
Age (years)	19~29	64/295 (21.7)	1.205	.272^b^
	30’s	89/375 (23.7)		
	40’s	114/411 (27.7)		
	50’s	68/250 (27.2)		
	≥60	43/184 (23.4)		
Education	≤ High school	240/958 (25.1)	0.014	.905^a^
	≥ College	138/557 (24.8)		
Resident areas	Large city	190/720 (26.4)	3.161	.075^b^
	Small city	155/623 (24.9)		
	Rural area	33/172 (19.2)		
Household income	<2,000	89/347 (25.6)	0.213	.644^b^
(1,000 won/month)	2,000 ∼ 2,999	109/406 (26.8)		
	3,000 ∼ 3,999	79/353 (22.4)		
	≥4,000	97/390 (24.9)		
Living style	Alone	26/76 (32.9)	2.834	.092^a^
	With family	351/1433 (24.5)		
Perception on ultimate beneficiary of CT	Pharmaceutical company	85/463 (18.4)	18.473	<.001^a^
	Hospital or physician	30/136 (22.1)		
	Patients	195/678 (28.8)		
	Advances in medicine	68/236 (28.8)		
Experience of CT participation	No	302/1114 (27.1)	4.131	.042^a^
	Yes	11/24 (45.8)		
Awareness of CT	No	65/377 (17.2)	15.929	<.001^a^
	Yes	313/1138 (27.5)		
Favorable feeling toward CT	Low	25/327 (7.6)	157.311	<.001^b^
	Moderate	128/680 (18.8)		
	High	225/508 (44.3)		
Safety toward CT	Low	28/410 (6.8)	240.003	<.001^b^
	Moderate	189/848 (22.3)		
	High	161/257 (62.6)		
Necessity toward CT	Low	2/87 (2.3)	63.434	<.001^b^
	Moderate	52/366 (14.2)		
	High	324/1062 (30.5)		

^a^Pearson *x* ^2^. ^b^Linear-by-linear association.

Multiple logistic regression was further conducted on the following six variables: perception of the ultimate beneficiary of CTs, experience, awareness, favorable feeling, safety, and necessity. All variables except for experience with CTs were significant predictors (Table 5). In multivariate analysis, the respondents who believed that the ultimate beneficiary of CTs was the patient or advances in medical science (rather than a pharmaceutical company) were more likely to participate in CTs (OR = 1.59, 95% CI 1.11–2.27, *p* = .011; OR = 1.60, 95% CI 1.02–2.52, *p* = .043, respectively). Respondents who were aware of CTs also were more willing to participate in CTs (OR = 1.82, 95% CI 1.35–2.45, *p* < .001). Participants with higher levels of favorable feeling, safety, and necessity for CTs were also more likely to be willing to participate. Those who were agreeable to participating in CTs had more favorable feelings toward CTs (moderate: OR = 1.70, 95% CI 1.00–2.89, *p* = .051; and high: OR = 3.25, 95% CI 1.91–5.54, *p* < .001), reported higher beliefs of CT safety (moderate: OR = 2.63, 95% CI 1.63–4.24, *p* < .001; and high: OR = 10.88, 95% CI 6.43–18.41, *p* < .001), and felt that CTS were more necessary (moderate: OR = 3.40, 95% CI 0.69-16.78, *p* = .133; and high: OR = 4.99, 95% CI 1.05–23.69, *p* = .043).

**Table 5:** **Factors associated with willingness to participate in clinical trials (N = 1,515)**

**Variable**		**Adjusted OR** ^**a**^	**95% CI**	***p***
Perception of ultimate beneficiary of CT	Pharmaceutical company^b^	1		
	Hospital or physician	1.562	0.84-2.91	.161
	Patients	1.589	1.11-2.27	.011
	Advances in medicine	1.600	1.02-2.52	.043
Experience with CT	No^b^	1		
	Yes	2.046	0.79-5.34	.143
Awareness of CT	No^b^	1		
	Yes	1.821	1.35-2.45	<.001
Favorable feeling toward CT	Low^b^	1		
	Moderate	1.698	1.00-2.89	.051
	High	3.254	1.91-5.54	<.001
Safety of CT	Low^b^	1		
	Moderate	2.631	1.63-4.24	<.001
	High	10.877	6.43-18.41	<.001
Necessity of CT	Low^b^	1		
	Moderate	3.40	0.69-16.78	.133
	High	4.99	1.05-23.69	.043

^a^Adjusted for other significant factors; obtained by multiple logistic regression analysis.

^b^Reference group.

## Discussion

The current study provides the first nationwide population-based data of Koreans’ awareness of CTs and willingness to participate in CTs. Awareness is the first step to the successful implementation of CTs. The main finding of this study was that willingness to participate in CTs in the future was affected by respondent awareness and perception of CTs, such as favorable feeling, safety and necessity of CTs.

The willingness to participate in CTs was significantly higher among respondents with awareness of CTs than it was in those who did not know about CTs. This result is consistent with previous reports [6,11]. Comis et al. [6] reported that respondents who had a high level of understanding of CTs were more likely to have a positive attitude toward participation than those with lower levels of understanding. These results show that the understanding of CTs is a cornerstone to the successful recruitment and retention of trial participants. Most of the respondents in the present study (86%) had a general idea about CTs from the mass media, such as TV or radio. Therefore, mass media campaigns could be a good strategy to increase the awareness of CTs, which was also suggested by previous researchers [11].

A strong association between positive perception (especially for safety and favorable feeling) and willingness to participate in CTs was found in this study. Participants who perceived CTs to be highly safe were approximately 11 times more likely to be willing to participate in CTs compared to those who were less sure of the safety. These results suggest that a fear of adverse effects from the drug or treatment is one of the most prominent barriers to participation in CTs. The issue of safety in CTs was also addressed in previous studies. Concern about drug side effects was the most important cause of unwillingness to participate in CTs [12], and recruitment remained a challenge for participants worried about being assigned to a less effective treatment [13]. Studies have also shown that a previous bad experience or mistrust in the process of obtaining informed consent or establishing a trusting relationship with their doctors is also a barrier to participation in CTs [12,14]. A reduction in trust as a result of knowledge of the Tuskegee Study, an example of a deliberately misleading CT that harmed some participants, has decreased the willingness of African Americans to participate in a medical research study [15]. Therefore, effective communication between participants and researchers is crucial to establish trust and facilitate CT participation. Researchers must make more efforts and dedicate time to offer balanced information between benefits and risks, and to explain the expected adverse reactions or disadvantages to participants, as well as to address the responses that would be taken in such situations. Most of all, adherence to research ethics, which provide investigator guidance for human rights protection in order to maximize research benefits, reduce risks and assure distributive justice to CT participants during trials, is the most important factor in the success of a CT [9]. Therefore, nurses need to be well-informed of related ethics and guidelines, as existential advocates for clients.

Although 75% of adult respondents were aware of clinical trials and perceived the high necessity of CTs, only 25% of participants reported willingness to participate in CTs in the future. These results are very similar to those of a study in Germany that reported that while 89.5% of survey participants judged CTs to be important, only 25% expressed willingness to participate [16]. In their study, the willingness to participate was significantly higher in people who thought CTs were important, had knowledge about CTs and had previously participated in CTs. In our survey, 25% of the respondents were willing to participate, which was somewhat lower than the results of other previous results, and may be an indication of differences in race, ethnicity or culture in the study participants [6,8,9]. A survey of 1,022 adults in England reported that the majority of respondents were willing to participate in CTs for major illnesses (63%) and cancer (65%) [17]. However, the lower rate of willingness to participate in our study increased about two-fold (48.6%) when participants were given a scenario in which their family member had a disease and needed a new treatment or drug. This finding may reflect that participation does not only depend on the perception or attitude of respondents, but also on other factors. Actual CT participation may be different from reported actions, especially when people are confronted with a family member’s diagnosis of illness. Various factors that were not measured in this study can affect actual participation rates, such as participant health status, comorbidities, availability of treatment options, economic benefit, participant burden, and inconvenience.

The likelihood of the general population to participate in CTs was not different by age, educational level or socioeconomic status in this study. However, residents of metropolitan cities, those who were male, and people in their 40s and 50s were more likely to participate in CTs. These results are interesting because a previous study indicated that a busy lifestyle, lack of time due to work and the existence of family were some barriers to participation in CTs [12]. In our study, younger adults were significantly more likely to have negative views on the necessity of CTs than were middle-aged adults, and younger people also had a negative tendency to participate in CTs. These results were different from those of a previous study [6], which reported that younger adults are more likely to have a positive perspective on participation in CTs than are older adults. However, our findings agreed with those of another study [18]. A literature review found altruism to be a major factor of CT participation among the general population [5]; weak altruism, which is an unwillingness to accept more than minimal personal risk for the sake of communal benefit, may hinder participation [19]. The younger generation in Asia grew up in an era of a conspicuous trend toward nuclear families, a rapidly developing economy and likelihood for adapting Western culture and values; these factors might have different effects on public altruism compared to those of the older generation. However, in concordance with earlier studies, associations between altruism and participation intention were also identified in the present study. Respondents who believed that the ultimate beneficiaries of CTs were the patients or advances in medical science rather than pharmaceutical companies were more likely to participate in CTs.

Previous experience with CT participation was associated with the willingness to participate in CTs in the univariate analysis, but this prior experience did not affect the likelihood of participating in CTs in this study. About 2% of our respondents had previously taken part in CTs. A very small fraction of previous CT participants among the respondents may mask a true difference in willingness in this study. Ohmann and Deimling [16] reported that previous participation in a CT was significantly associated with positive trial participation intention. Therefore, the strategy to share previous participant experience regarding the processes involved, the process of informed consent and individual or social benefits as outcomes of CTs with the general public through the mass media could have an impact on CT awareness and attitude toward CTs.

Recently, Korea Food and Drug Administration (KFDA) had announced the roadmap to foster the growth of CTs toward a leading country in participating in biopharmaceutical CTs including a strategy to enhance communication to the general public on CTs. As a part of this, the Korea National Enterprise for CTs (KoNECT) distributed a 4 minute movie enhancing awareness of and necessity for CTs to any institution with the educational purpose for patients or the general public. We expect that the mass media campaign to improve public awareness and trust on CTs would be carried out in the near future in Korea. However, a mass media campaign itself might not guarantee the increment of patient willingness to participate in and accrual to CTs [20]. The higher enrollment rates could be achieved through positively changing patients and their family’s attitude toward participation in CTs using more targeted educational approaches [21].

Strengths of this study included the use of a sufficiently large probability sample to represent a nationwide population and to compare the differences among geographical regional area within 5% of the sampling error. However, this study had several limitations. First, willingness to participate in CTs does not reflect actual enrollment: only a behavioral intention. Respondents may be more likely to answer positively about willingness to participate due to a tendency to exhibit pleasing behavior. Future studies should examine the extent to which behavioral intention predicts actual enrollment in CTs and also the circumstances under which participation does not occur. Second, the willingness to participate in CTs depends on factors other than the attitude of the patient. Therefore, various factors which were not measured in this study can affect the actual participation rates in CTs. Nonetheless, findings from this study can be useful in understanding the willingness of Asians to participate in CTs.

## Conclusions

Our study results illustrate the current levels of awareness and perception toward CTs in Korea and also clarify the important factors that may predict a person’s willingness to participate in CTs. These results indicate that an association among awareness, perception toward CTs and willingness to participate is a result of differential perception on issues related to trust of CTs. A better understanding of the perspectives of members of the general public who are potential participants in a future CT would likely improve recruitment. These findings might be helpful for improving clinical researchers’ understanding about their participants and useful when developing effective outreach strategies for recruitment and retention for CTs.
